# The Organization of Local and Distant Functional Connectivity in the Human Brain

**DOI:** 10.1371/journal.pcbi.1000808

**Published:** 2010-06-10

**Authors:** Jorge Sepulcre, Hesheng Liu, Tanveer Talukdar, Iñigo Martincorena, B. T. Thomas Yeo, Randy L. Buckner

**Affiliations:** 1 Howard Hughes Medical Institute, Cambridge, Massachusetts, United States of America; 2 Department of Psychology and Center for Brain Science, Harvard University, Cambridge, Massachusetts, United States of America; 3 Athinioula A. Martinos Center for Biomedical Imaging, Department of Radiology, Charlestown, Massachusetts, United States of America; 4 European Bioinformatics Institute, Cambridge University, Cambridge, United Kingdom; 5 Department of Psychiatry, Massachusetts General Hospital and Harvard Medical School, Boston, Massachusetts, United States of America; Indiana University, United States of America

## Abstract

Information processing in the human brain arises from both interactions between adjacent areas and from distant projections that form distributed brain systems. Here we map interactions across different spatial scales by estimating the degree of intrinsic functional connectivity for the local (≤14 mm) neighborhood directly surrounding brain regions as contrasted with distant (>14 mm) interactions. The balance between local and distant functional interactions measured at rest forms a map that separates sensorimotor cortices from heteromodal association areas and further identifies regions that possess both high local and distant cortical-cortical interactions. Map estimates of network measures demonstrate that high local connectivity is most often associated with a high clustering coefficient, long path length, and low physical cost. Task performance changed the balance between local and distant functional coupling in a subset of regions, particularly, increasing local functional coupling in regions engaged by the task. The observed properties suggest that the brain has evolved a balance that optimizes information-processing efficiency across different classes of specialized areas as well as mechanisms to modulate coupling in support of dynamically changing processing demands. We discuss the implications of these observations and applications of the present method for exploring normal and atypical brain function.

## Author Summary

Information processing in the human brain arises from both interactions between adjacent brain areas and from distant projections that form distributed systems. Here we estimated functional connectivity profiles in the human brain using a novel approach to map the regional balance between local and distant functional connectivity. We discovered that the human brain exhibits distinct connectivity profiles across regions with primary sensory and motor areas displaying preferential local connectivity and heteromodal association areas displaying preferential distant connectivity. These findings expand our knowledge of how the human brain has specialized its architecture to optimize processing efficiency and provides an approach to measure, in individuals, the degree to which the typical balance of local and distant connectivity is present.

## Introduction

The human brain is a complex biological structure with specializations for local, modular processing that are distinct from anatomical properties that facilitate integrative processing. Specifically, anatomic projection patterns suggest a division between areas that form domain-specific hierarchical connections [1]–[4] and distinct heteromodal association areas that receive widespread projections from distributed brain systems [5]–[9]. The dichotomy is not absolute. Sensory systems contain divergent projections and display multimodal convergence at advanced processing stages. Nonetheless, dominance for one connectivity profile over the other is present for many areas and suggests a fundamental organizing principle of cortical-cortical connectivity. Early sensory cortical areas are examples of areas with predominantly local hierarchical connections (e.g., see [2]) while prefrontal, lateral temporal, limbic and paralimbic areas form hubs linking widely distributed connections – neural epicenters of large-scale distributed networks [8].

Studies of comparative anatomy suggest that the ratio of local to distributed areal projections may be critical to the evolution of higher-order cognitive functions including language, reasoning, and foresight. The hominin brain has tripled in absolute size over the past 2–3 million years including a disproportionate enlargement of cortical surface area [10], [11]. However, expansion comes with a cost to information processing efficiency [11]. Proliferation of long-distance connections and increasing brain volume could lead to untenable wiring lengths if they evolved unchecked [12]. Thus, there is a compensatory pressure to modularize information flow within parallel processing pathways and to maximize efficient communication among areas of similar function. Van Essen [13] proposed that there is a specific selection pressure to optimize wiring length between adjacent functionally-similar areas within the same hemisphere. Consistent with this possibility, cortical folding patterns in the macaque brain minimize between-area wiring lengths for sensory (e.g., Broadmann's area [BA] 17 to BA 18) and motor (e.g., BA 4 to BA 6) pathways.

The relative proportion of association cortex differs further in the human [14],[15]. The human brain is three times larger than that of modern great apes yet primary motor (BA 4) and visual (BA 17) cortices are about the same absolute size [16], [17]. Preuss [14], [18], in a detailed analysis of cortical growth, concluded that widely distributed associated areas exhibited an increase in absolute surface area during hominin evolution including higher-order parietal and temporal areas as well as prefrontal cortex. Thus, the long-held belief that the prefrontal cortex is preferentially expanded in humans is only partially correct; heteromodal association areas are likely expanded throughout cortex including those areas falling within prefrontal cortex. Bolstering these observations, surface-based analysis of cortical differences between macaque and human based on 23 estimated homologous areas reveals a high degree of expansion in parietal, lateral temporal, and dorsolateral prefrontal regions and a relative compression of sensorimotor and visual areas [19].

The modern human brain also possesses a high proportion of cerebral white matter relative to contemporary primates including the great apes [20], [21] (see also [22] for a broad analysis of primates). Comparative study of the arcuate fasciculus, the major fiber bundle connecting anterior and posterior heteromodal language zones, shows that it is enlarged in humans as compared to chimpanzees or macaques [15]. Thus long-distance association projections have expanded as well and may have done so in relation to specific functional adaptations. One can presume that there has been considerable pressure to maintain efficient wiring and network properties as the complexity of cortical connectivity and association cortex has increased, especially considering long-distance projections are well represented in the human brain (see [23]).

All of these findings converge to suggest that the balance between long-range projections and local areal interactions is important for efficient cortical processing. While this balance has been recognized for some time (e.g., see [5], [7], [8]), recent computational explorations of connectional patterns have brought the issue into sharp focus [24]. Graph theory, in particular, provides informative metrics to analyze properties of complex networks [25]–[31]. When applied to the study of connectional anatomy, analyses consistently reveal that cortical networks exhibit “small world” properties [32], [33]. Connections are not randomly dispersed among cortical areas but rather show strong clustering patterns and hubs that allow for relatively short path lengths to propagate information through the networks [34].

Moreover, the extent to which an individual area is central to maximizing communication between multiple areas can be quantified and cortical regions possessing hub-like properties can be mapped. Applying this analysis strategy to structural [35] and functional [36], [37] human connectivity data reveals a core set of regions along the cortex including paralimbic areas and parietal association areas that behave as hubs. The resulting map of these regions in humans includes the many known heteromodal association areas spread throughout prefrontal, parietal, and lateral temporal cortex and bares a strong resemblance to the estimated regions of cortical expansion in human as compared to macaque (e.g., contrast [37] with [19]).

Although previous studies have focused their attention in network topological modularity [38]–[41] and in some aspects of the relationship between physical distance and connectivity [29], [36], [42], connectivity profiles that differentiate local and distant projection patterns have not been fully characterized. Physical distance and network path length, as discussed above, are among of the most central properties to efficient information propagation.

There are two likely reasons for this omission. First, human studies using diffusion techniques to measure anatomic connectivity (diffusion tensor imaging; DTI) provide poor information about connectivity between areas that are supported by local association fibers (u-fibers) and neighborhood association fibers that connect immediately adjacent and nearby areas [43]. Commonly used diffusion imaging techniques capture long association fibers that travel in discrete fascicles within the hemisphere and commissural fibers that pass between the hemispheres (but see [44] for a recent exception), and usually discard fibers or fail to adequately measure information from close or adjacent regions.

Second, functional connectivity approaches that measure cortical-cortical interactions indirectly via correlated blood oxygenation level-dependent contrast (BOLD) [45]–[47] have not focused on local anatomic correlations because of the relatively poor spatial resolution of the approach. While the blood flow response is locally regulated (under certain conditions at the level of the cortical column; e.g., [48]), the current practical resolution for exploring large cortical regions is about 3–4 mm [49]. This makes exploring within-area lateral connections challenging. However, the achievable resolution of functional MRI (fMRI) is well within the expected resolution needed to provide information about adjacent and nearby areas that are distinct from interactions carried by long association fibers and other long-range connections. Measurements at this intermediate resolution should be rich in information about the connectional architecture of the human brain including information about whether cortical areas possess local modularity.

**Figure 1:**
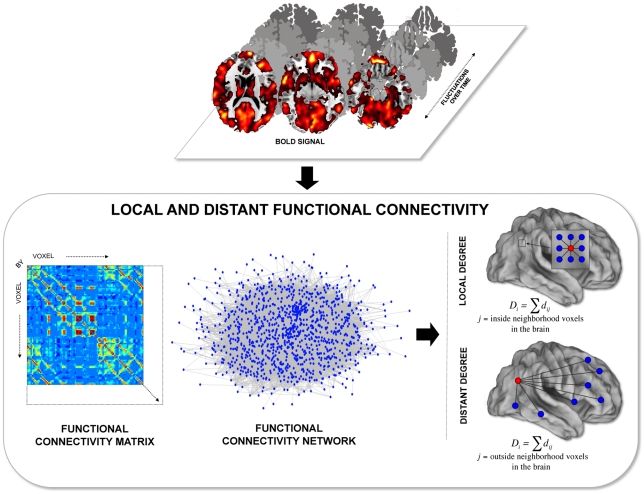
Methods for identifying local and distant functional connectivity. The basis of the present method is the intrinsic BOLD signal fluctuations that correlate between brain regions. The functional connectivity matrix was computed to represent the strength of correlation between every pair of voxels across the brain; the pattern of these connections is the functional connectivity network. The displayed example represents a matrix and network of 1000 nodes (brain voxels). To estimate local and distant brain connectivity, the normalized degree of intrinsic functional connectivity of every voxel across the brain was computed taking into account physical distance to compute separate estimates of local degree connectivity and distant degree connectivity (see also Figure S1).

Motivated by this possibility, we developed and applied a novel approach to map the regional balance between local and distant functional connectivity in the human brain. We first extended a computationally efficient approach based on network graph theory [37] to map the degree of intrinsic functional connectivity between regions throughout the brain, taking into account the local neighborhood connections as well as the remote or distant connections (within and outside 14 mm of a neighborhood area) (Figure 1). Control analyses showed that the method successfully and reliably identified distinct local degree values across the brain. Estimates of these values were then used to explore the properties of regions across the brain and to compare these estimates to those derived from well-known network measures including path length, physical cost, and clustering coefficient. Finally, we examined functional connectivity during an active task (as contrast to rest) to examine how functional coupling dynamically changes in response to task demands.

## Results

### The Human Neocortex Displays a Complex Topography of Preferential Local Versus Distant Connectivity

**Figure 2:**
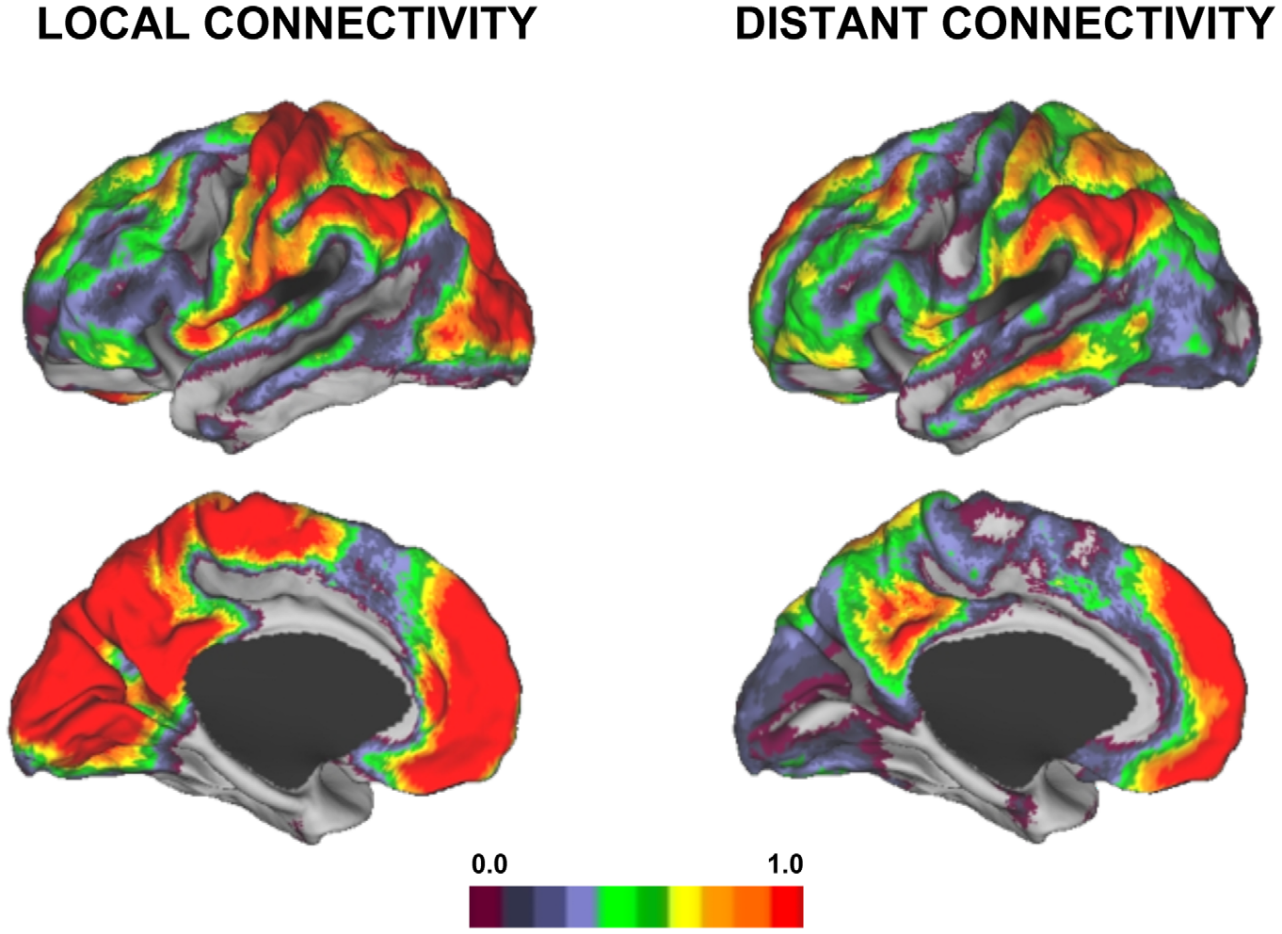
Local and distant functional connectivity maps. Local (left) and distant (right) functional connectivity maps are displayed for the left hemisphere from 100 subjects. Data were acquired during passive (rest) fixation. Notable differences in the topography of the connectivity profiles are present with primary sensory and motor regions showing strong local connectivity and regions of association cortex displaying distant connectivity (see also Figure S3). The surface projection uses the PALS approach of Van Essen (2005; see text). The color bar represents the normalized degree connectivity (Z-score).

**Figure 3:**
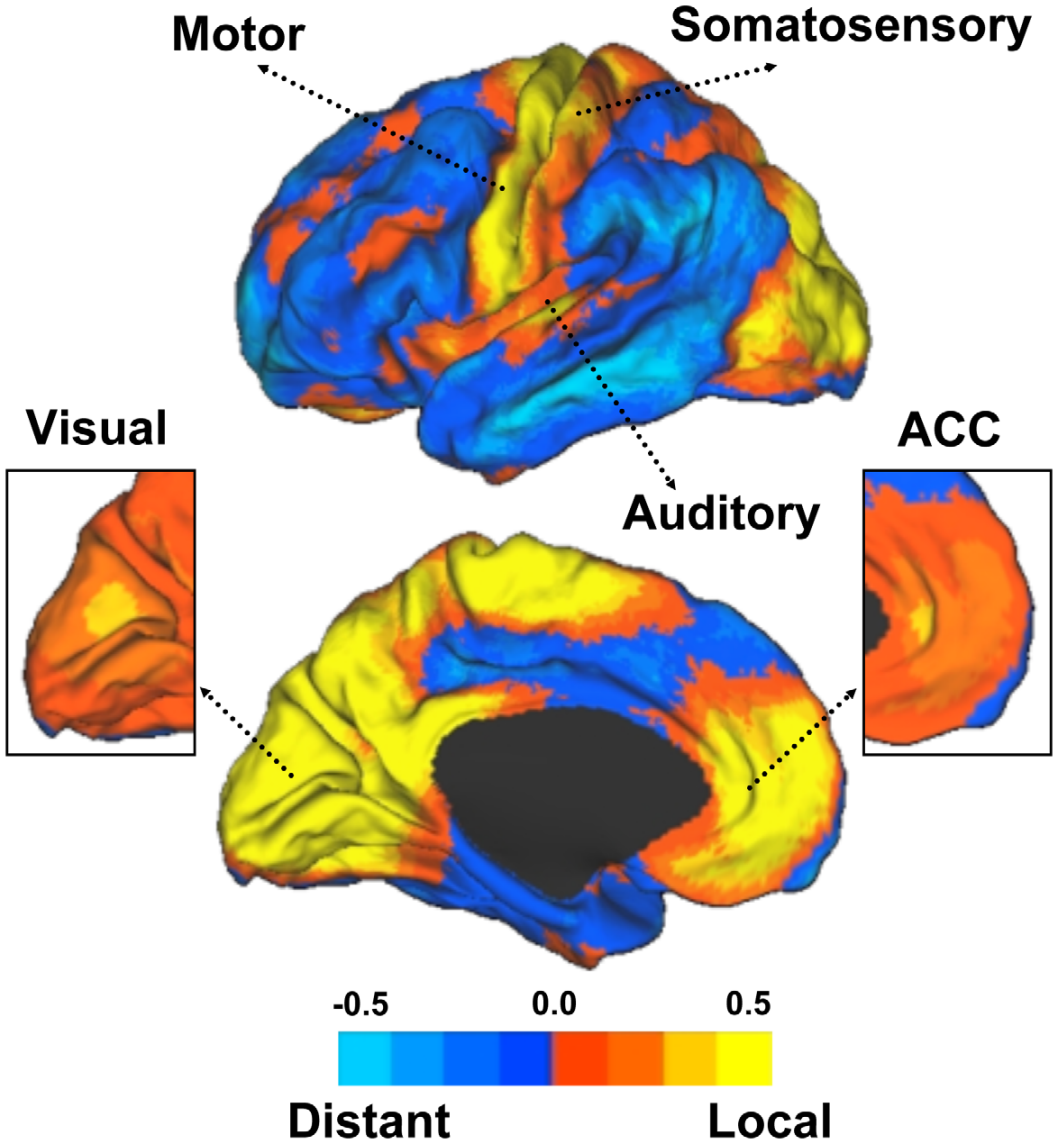
Preferential connectivity maps highlight regions with differential functional connectivity profiles. Data from Figure 2 are contrast to illustrate the relative differences between local and distant degree connectivity. The maps plot the direct subtraction of distant versus local functional connectivity with blue indicating regions of preferential distant connectivity and yellow indicating regions of preferential local connectivity. Note that the primary sensory and motor regions show different profiles as contrast with association cortices in the parietal, temporal and frontal lobes. The insets display the same maps but at an increased threshold to appreciate the high relative local degree connectivity in visual cortex and rostral anterior cingulate (see also Figure S3). ACC = anterior cingulate.

**Figure 4:**
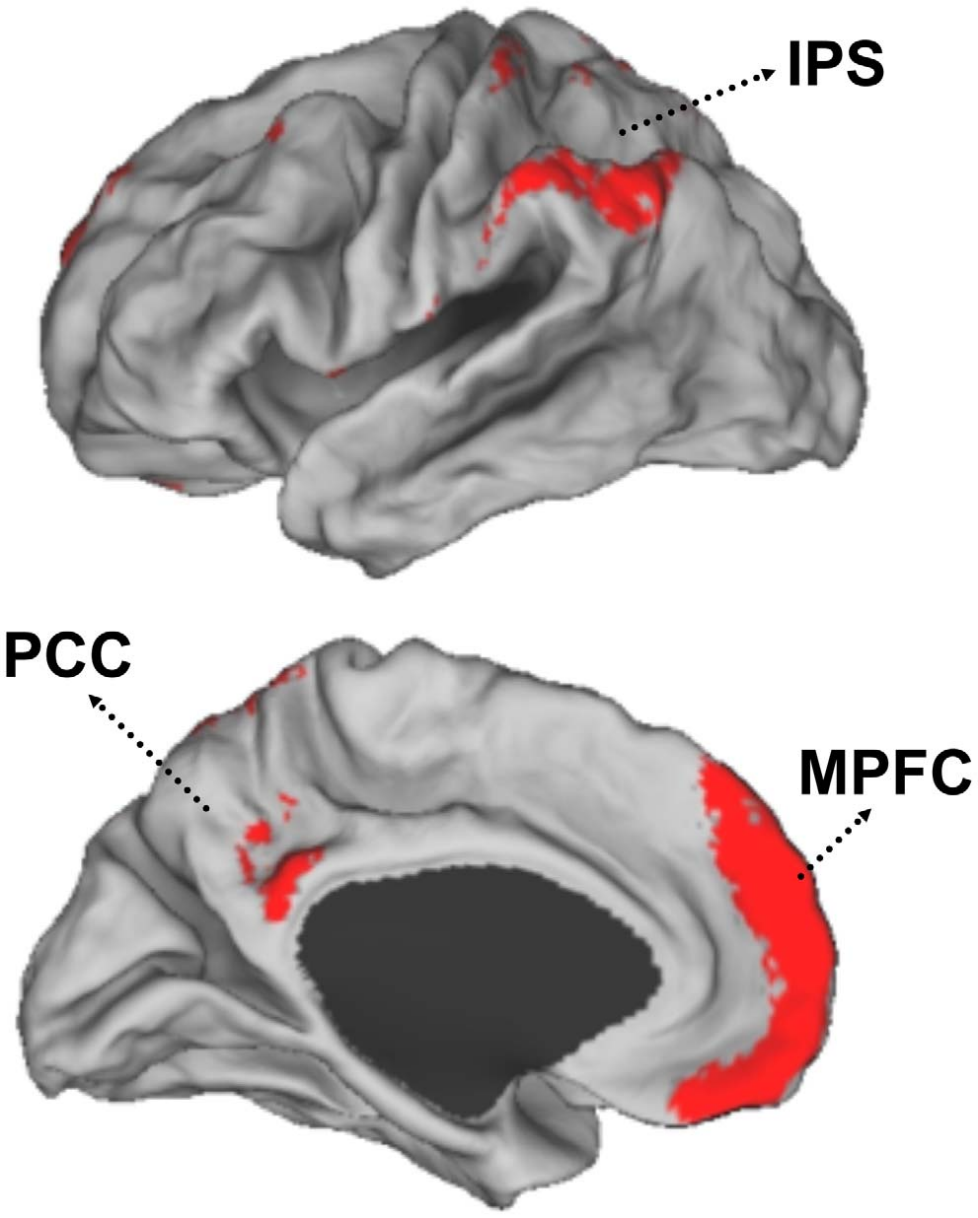
Some regions display both high local and high distant functional connectivity. Plotted in red, using the same format as Figure 2, are regions that show both high local and distant functional connectivity (normalized degree cutoff of Z-score>1.0). These regions include the posterior cingulate cortex (PCC), a region ventral to the intraparietal sulcus (IPS) extending into the inferior parietal lobule, and the medial prefrontal cortex (MPFC). Note that the region within the MPFC does not extend into the rostral ACC that displays a preferential local connectivity profile (see Figure 3).

Local and distant functional connectivity are plotted separately (Figure 2) as well as combined into maps of preferential connectivity (local – distant; Figure 3) and overlap (local ∩ distant; Figure 4). Based on these maps regions could be characterized into three broad categories: 1) Regions displaying preferential local connectivity with less distant connectivity, involving mainly primary and secondary/modality-selective cortices (motor, somatosensory, auditory, visual, and a region at or near the supplementary motor area [SMA] proper), 2) Regions displaying preferential distant connectivity with relatively low local connectivity including heteromodal areas in the lateral parieto-temporal and frontal cortices, and 3) Regions that contained both a high degree of local and distant connectivity including prominent midline regions that comprise components of the default network (posterior cingulate, certain regions within the medial prefrontal cortex). The third connectivity profile is most clearly visualized by examining the overlap of the maps (Figure 4). Figure S1 and S2 display maps at several levels of threshold and left/right projections to illustrate that the topographies of the preferential and overlap maps are qualitatively consistent across thresholds. Volume displays of the maps are also provided for transverse sections in the atlas space of the Montreal Neurological Institute (MNI) (Figure S3). The full volume data are available from the authors upon request.

**Figure 5:**
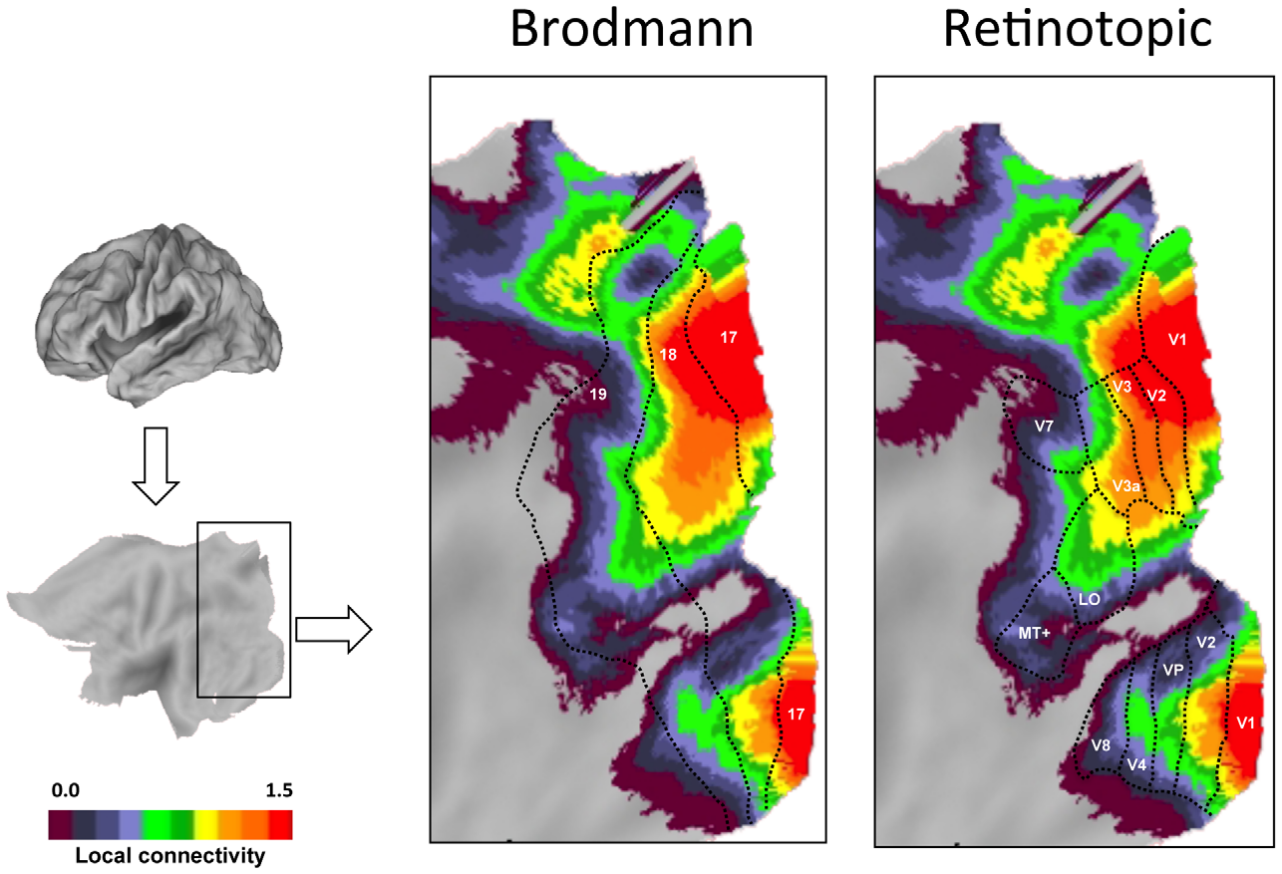
Retinotopic visual areas display preferential local functional connectivity. Preferential functional connectivity data (from Figure 3) are plotted in relation to approximate Brodmann areas (left) and estimated retinotopic boundaries (right) for visual cortex. The display represents a flattened portion of cortex that includes the occipital lobe. Labels in the Brodmann panel represent approximate Brodmann area boundaries (see text). Labels in the Retinotopic panel represent estimated visual areas (see text). The regions of high preferential local connectivity fall within the early retinotopic areas including primary visual cortex.

**Figure 6:**
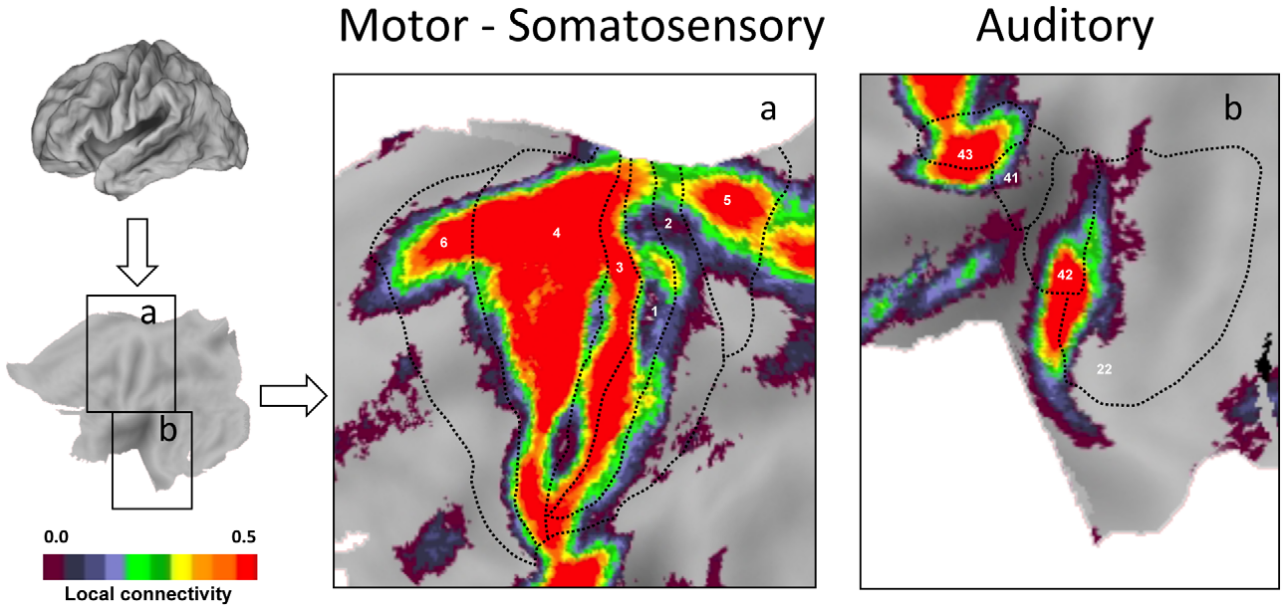
Somatosensory, motor and auditory areas display preferential local functional connectivity. Preferential functional connectivity data are plotted in relation to approximate Brodmann areas for somatosensory/motor cortex (left) and auditory (right) cortex. Labels represent Brodmann areas.

A striking feature of the maps is that regions near primary sensory and motor cortex show strong preferential local connectivity. Examining the topography of the regions in more detail revealed that they track estimated boundaries of primary sensory and motor areas (Figures 5 and 6). For example, the regions of the visual system that show strong preferentially local connectivity overlap well with the early retinotopic areas that extend from V1 to V3a and V4 (Figure 5). In this regard, the analytic procedure of mapping local versus distant functional connectivity at rest is sufficient to reveal the well-established distinction between primary/secondary and association cortices. Regions with high distant degree connectivity and high local degree connectivity converged on multiple regions that fall within the default network [50], [51].

### Does Local Functional Connectivity Change During Task Performance?

**Figure 7:**
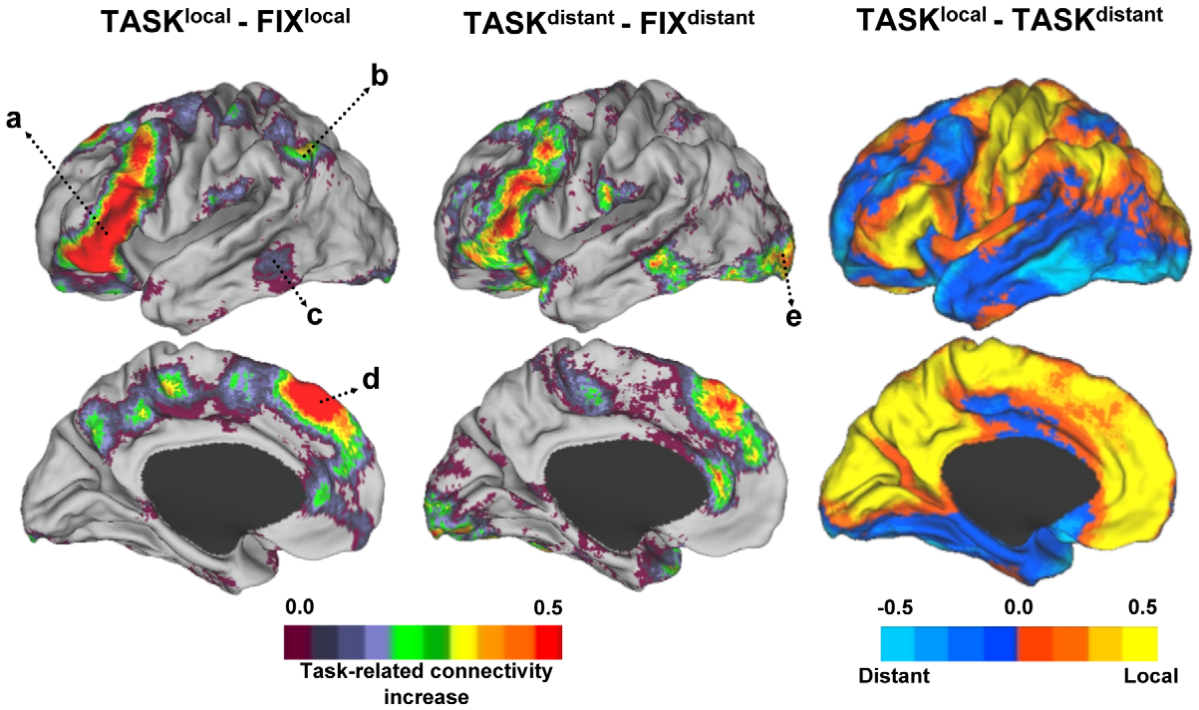
Task performance leads to changes in local and distant functional coupling. Changes in the degree of functional connectivity during performance of a continuous semantic classification task are displayed for local connectivity (left) and distant connectivity (center). An increase in local functional coupling is observed along the inferior frontal gyrus (a), the inferior parietal lobule (b), lateral temporal cortex (c), and the dorsal anterior cingulate (d). More modest (but anatomically similar) increases in functional coupling are noted for distant functional coupling with the exception of visual cortex (e) that shows a more prominent change in distant functional coupling. Despite the task differences, data acquired during rest fixation and continuous task performance show similar locations of preferential local connectivity within motor and sensory regions (right).

Although functional connectivity patterns measured at rest provide valuable information about the intrinsic architecture of the brain, they are not synonymous with anatomic connectivity and are influenced by the task state (see [47] for recent review). For this reason, we next explored the influence of task performance on local and distant connectivity. Two results emerged (Figure 7).

First, engaging the task influenced both local and distant functional connectivity in regions typically active during performance of the abstract/concrete classification task. The changes were particularly prominent in the local connectivity estimates and included prefrontal cortex along the inferior frontal gyrus, lateral temporal cortex, dorsal anterior cingulate and a posterior parietal region linked to the frontal-parietal control system (e.g., [52]). Thus, one unexpected observation is that local functional connectivity can be used to measure engagement of task regions in a manner that is distinct from previous approaches to fMRI data analysis. A subtle change was also noted in increased (relative) distant connectivity in visual regions perhaps reflecting coupling of sensory regions to association areas during task engagement.

Second, the regions of preferential local functional connectivity, as revealed by the direct contrast of the local to distant connectivity maps obtained from the task data, included the primary sensory and motor cortices (Figure 7; right column). Inspection of the data in reference to cortical flattened representations once again showed that the strongest preferential local connectivity estimates were within or near early retinotopically-defined visual areas. That is, despite some relative changes in local and distant functional coupling during the task state, sensory areas still persisted in having preferentially local connectivity profiles.

### Relationship to Network Measures of Path Length, Physical Cost, and Clustering Coefficient

**Figure 8:**
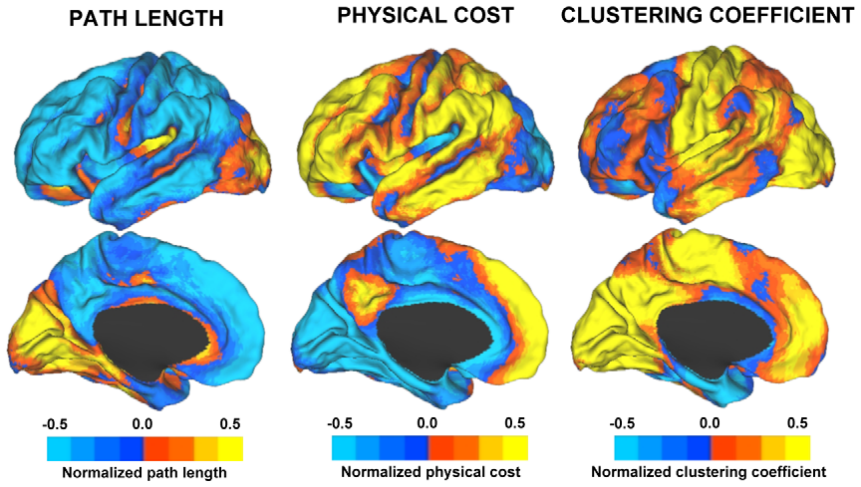
Maps of path length, physical cost and clustering coefficient. Map estimates of path length (left), physical cost (center) and clustering coefficient (right) are displayed. Compared to the local-distant preferential map (Figure 3 and Figure S1), short path lengths and high levels of physical cost are predominant in regions of preferentially distant connectivity. Preferential local connectivity is associated with regions of high path length, low physical cost and high clustering coefficient.

To situate our findings in the context of other well-known network measures, we computed the average path length, physical cost and clustering coefficient in our data set (Figure 8). Average path length is a measure of how far a node is, on average, from all other nodes in the network. Low path lengths (blue in our scale in Figure 8; left column) are those regions that have the shortest path lengths to other regions of the brain. Physical cost reflects, in some sense, the opposite property and plots, in our scale, regions with physically distant connected regions in yellow and orange. Clustering coefficient is a measure of segregation and, in our scale, displays regions with the greatest level of local modular organization in yellow and orange.

As shown in Figure 8, regions with preferential local connectivity fall within regions that are characterized by long path length, low physical cost and high clustering coefficient. Low levels of network topological path lengths and high levels of physical cost are prominent in regions of distant preferential connectivity. This relationship is perhaps most apparent when comparing the local degree map in Figure 2 and the clustering coefficient map in Figure 8. It is also possible to detect differences between the local/distant preferential map (Figure 3) and the network measures (Figure 8).

### Reliability and Control Analyses

The primary results of our analyses are the map estimates of local and distant functional connectivity. Several parameters were set to complete the analyses (e.g., the distance threshold) and therefore processing decisions may have affected the results. A series of control analyses were conducted to boost confidence in the approach and to establish that the reported results are robust. First, test-retest reliability was assessed for the local and distant connectivity maps by comparing maps derived from two independent datasets each comprising 50 participants (Figure S4). High correlation coefficients between the two samples were obtained (r = 0.95 for local and 0.91 for distant degree connectivity). Next, the influence of changing the distance threshold on the resulting local connectivity maps was examined by varying the neighborhood from a radius of 6 mm to 18 mm (Figure S5). The radius of 6 mm yielded a map that did not notably distinguish areal topography consistent with the limited spatial resolution of the technique. Results showed largely stable estimates of local connectivity for neighborhood radius values greater than 10 mm. We conservatively used a distance threshold of 14 mm for all analyses. The influence of Gaussian smooth was examined by comparing maps without spatial smoothing to the chosen 4 mm full-width half-maximum (FWHM) smoothing kernel (Figure S6). Removing the spatial smooth did not qualitatively affect the results; however, the preferential effects in the degree maps were generally less robust consistent with a reduction in signal-to-noise ratio.

We further examined whether correlations between the hemispheres across the midline contributed to the observed results. Bilateral contributions might cause a bias in overestimating local connectivity values along midline structures. Maps that included degree connectivity for only one hemisphere were highly similar to those that included both hemispheres (Figure S7). Masking the cortex to include only the cortical mantle (excluding subcortical regions including the basal ganglia, thalamus, and midbrain as well as the cerebellum) also did not qualitatively change the results but did lead to several subtle differences presumably arising from exclusion of distant thalamic, striatal, and cerebellar connections (Figure 8). As a final exploration we examined the influence of the specific normalization approach and also the effect of grey matter volume correction (Figure 8). Again, the results were largely robust to analysis variations.

## Discussion

The present study applied a novel approach to analyze regional functional connectivity profiles taking into account the distance between correlated regions. We found that the human brain exhibits cortical functional connectivity profiles at rest that fall into three major categories: one for sensory and motor cortical regions (preferential local connectivity), one for many regions near heteromodal association areas (preferential distant connectivity), and one related to a subset of heteromodal association and paralimbic regions that fall along the midline, in regions that are core components of the default network (high local and high distant connectivity). Specifically, preferential local and distant connectivity profiles revealed that regions within or near primary sensory and motor areas display high local connectivity consistent with a modular organization. By contrast, distant connectivity is prominent across association areas in parietal, lateral temporal, and frontal cortices as well as paralimbic cortex including posterior cingulate. These regions have been previously described as important for higher-order cognitive functions such as attentional, memory and language processes. Among the set of regions with a high degree of distant connectivity is a subset that simultaneously possess both high local and distant connectivity. The presence of densely interconnected local (modular) systems linked by connectional hubs is indicative of complex systems that gain efficiency through “small world” properties [24], [29], [30]. We discuss the implications of these connectional profiles and applications of the present method for understanding neuropsychiatric illness.

### Regional Differences in Local Connectivity Profiles

A strong expectation that human primary sensory cortex will possess properties consistent with a high local distance connectivity organization is provided by prior analyses of macaque anatomic connectivity (e.g., [2]). The absence of methods able to measure local anatomical connectivity, including detecting small u-fibers and local association connectivity, has limited the ability to visualize this basic organizational property. Our results demonstrate that differences in connectivity profiles were prominent across the cortex. Figure 3 summarizes these differences and Figures 5 and 6 provide detailed examination of sensory and motor areas. Regions that encompass estimated boundaries of early sensory and motor areas display high levels of local connectivity, most likely as a result of local anatomical connections between adjacent or nearby areas. Within the visual cortex, the region with the most pronounced local connectivity organization is at or near V1 with a gradual transition to less modular connectivity profiles as one moves along the hierarchical progression from V2 through V3a, supporting a functional gradient. Regions anterior to the estimated boundary of the MT+ complex display preferentially distant connectivity.

Regions at or near primary somatosensory, auditory cortex, and motor cortex also display high levels of local connectivity. Some of the details of the mapping are not fully aligned with expectations, particularly the dense local connectivity observed in area 43 but not area 41 in auditory cortex. It is presently unclear whether this is a differential property of the area, an inaccuracy of the present method, or a consequence of inaccurate estimates of the areal boundaries. Of the regions studied, the best alignment between expectations from macaque anatomy and estimated human areal boundaries was for the retinotopically mapped visual areas [53]. Equivalent mapping in human auditory cortex was not available. Thus, an important future direction will be to map sensory areas within individual subjects and explore further the correspondence between areal boundaries and connectivity profiles.

We also found that certain regions along the midline, including portions of the anterior cingulate, have preferential local connectivity and less distant connectivity similar to somatosensory/motor, auditory or visual cortices (see inset detail in Figure 3). In humans the portion of cingulate just anterior to the genu of the corpus callosum includes areas 24 and 32ac, possible homologues to macaque areas 24a/b/c [54], [55]. In their seminal studies of medial prefrontal cortex in the macaque, Carmichael and Price [56] noted that areas 24a/b, 10m and 32 are tightly interconnected components of the “medial network”. Unlike other areas of high local connectivity, areas 24a/b do not receive significant projections from sensory systems (barring some olfactory inputs to the region). Inputs are predominantly limbic. What is clear from the connectivity profiles is that cingulate cortex just anterior to the corpus callosum shows a markedly different connectivity profile than posterior cingulate and more anterior medial prefrontal regions that include human area 10. These regions, as will be discussed in the next section, show among the highest levels of both local and distant connectivity.

### Certain Regions Possess Hybrid Local and Distant Connectivity Profiles

Certain regions were estimated to simultaneously possess both high levels of local and distant connectivity (Figure 4). The posterior cingulate, medial prefrontal cortex, and inferior parietal lobule were the three most extreme examples of the hybrid connectivity profile. The importance of these regions as connectional hubs, in particular the posterior cingulate, has been noted previously based on anatomical [57], [58], diffusion [35], [59], and functional [37], [50], [60] connectivity data. Prior functional connectivity analyses using graph theory have previously revealed that these regions are hubs of long-distance cortical-cortical interactions with both high degree and betweeness centrality [36], [37]. What is new here is that a subset of these regions also possess among the highest levels of local functional connectivity.

The regions displaying the hybrid connectivity profile overlap regions that belong to the default network [50], [51]. This network has been implicated in cognitive functions associated with internal thought as contrast to stimulus-based perception. For example, the network is active during autobiographical memory retrieval [61], imagining the future [62], and mind wandering [63] (see [50], [64] for reviews).

These observations raise two questions: What are the functional consequences of a hybrid functional connectivity profile and why is it so prominently represented in the default network? While there are too few constraints to offer more than broad speculations, it is worthwhile to generate hypotheses to encourage further exploration. Given that the regions possessing hybrid connectivity profiles are active during internal modes of thought, including during passive fixation, it is intriguing to hypothesize that information processing that persists independent of strong sensory constraints might require a set of modular, tightly coupled areas to maintain efficient local processing. That is, the default network may possess features so that information processing is able to maintain stable in situ information (high local network connectivity) and simultaneously to associate distributed information from key limbic, parietal and prefrontal regions of the brain (high distant network connectivity). This idea extends what has been previously articulated. For example, in Mesulam's foundational work on transmodal areas [6]–[8], he emphasizes their role as pointers to distributed modular systems. The present hypothesis expands this notion to also explore processing contributions that arise directly from extensive local connectivity between and within certain contiguous association areas.

### Effects of Task and the Potential Use of Functional Coupling to Estimate Task Effects

An interesting unexpected result was the detection of clear local functional coupling changes when a task was engaged (Figure 7). Most of our analyses were of resting-state fixation data similar to the approach common to the literature. When a continuous task involving semantic classification of words was engaged, the preferential local connectivity profiles in sensory and motor regions were largely retained with the addition of strong local coupling within many of the distributed regions typically activated by the task. In fact, the map of increased local functional connectivity in the task state relative to rest fixation (Figure 7; left column) could easily be mistaken for a typical blocked-task functional MRI or positron emission tomography (PET) paradigm (e.g., compare our Figure 7 with Supplementary Figure 3 in [65]).

There are two separate implications of this observation. First, the result is a reminder that low-frequency functional correlations as measured by BOLD contrast fMRI are not an exact proxy for anatomical connectivity. Coupling among regions changes as a function of task state. Second, measures of local functional coupling may provide a means to investigate regional activity levels during tasks. It is possible, although not explicitly tested here, that local functional coupling may provide a powerful approach for identifying task-activated regions including for brief epochs of task performance. It will be interesting in future studies to explore this possibility and to generally examine what can be learned from task-induced changes in local functional coupling. A radical possibility is that task contrasts will not be required and local functional coupling by itself, or referenced to other aspects of the data or normative data, will be sufficient to estimate properties of brain activity.

### Limitations, Caveats, and Future Directions

The present work maps relative connectivity profiles that distinguish local and distant functional connectivity. As such it moves into an arena that is at the resolution boundary of present functional imaging approaches. Further, the connectivity method applied was based on intrinsic activity correlations measured using BOLD contrast fMRI. As we have discussed previously [47], there are strengths and limitations of this indirect approach for estimating connectional anatomy. Relevant here is the observation that intrinsic activity correlations can reflect more than direct monosynapatic connections including contributions of polysynapic projections and common driving inputs, such as from the thalamus. Our results also reveal clear, task-dependent modulation of functional coupling. Thus, it will be important to employ convergent methods to validate the anatomic properties that we infer. Diffusion-based methods that employ high angular resolution diffusion (e.g., DSI) may soon be able to map u-fibers and local association fibers [44]. To make a reasonable assessment of whether the properties we are observing reflect an artifact of smoothing or other processing steps, or the boundaries of functional resolution, we conducted a large number of control analyses (see Supplementary Figures S1, S2, S3, S4, S5, S6, S7). The core results are reliable and robust across alternative smoothing and processing decisions. Thus, while we anticipate further refinement of the methods, we expect that the main results of the paper are valid.

One potential limitation of our study is that we are approximating real cortical distance with Euclidean distance. In the case of nodes in adjacent gyri, the Euclidean distance is underestimating the true cortical distance, since the white matter tracts often bend around the intervening sulcus. Future work will be needed in order to include additional information regarding surface distance and ideally white matter tract path lengths (to approximate this approach see an example in [66]).

A further limitation of the present paper is the focus on group data. An important avenue for future work will be to explore these properties, perhaps even at higher spatial resolution, in the context of task-based estimates of functional areas. For example, it will be useful to explore whether individual differences in estimated boundaries of retinotopic visual areas track estimates of local distance connectivity. We predict they will. Similarly, the regions of preferentially distinct connectivity overlap with regions that are active during tasks of remembering, foresight, and other forms of high-level cognition [50], [64]. It will be important to study more explicitly the overlap between connectivity profiles and activity during these forms of cognitive task at the individual subject level.

Exploration of individual differences has potentially important implications for study of genetics and neuropsychiatric illness (e.g. Alzheimer's disease, epilepsy, schizophrenia, bipolar disorder, and autism). A particularly interesting area of future exploration concerns the development of local and distinct connectivity and the relation of our metrics to atypical development. Certain neuropsychiatric disorders are suspected to result from molecular disruptions that give rise to aberrant connectivity patterns. For example, autism is associated with overgrowth of the brain early in development and adult white-matter abnormalities [67]. Atypical development may affect the fragile balance between local and distant connectivity.

Relevant to this possibility, Fransson and colleagues [68] demonstrated functional connectivity in infants at typical birth age (the infants were preterm). They found strong connectivity among sensorimotor networks but did not identify connectivity within the association networks typical of adults, including the default network. Fair et al. [69] reported that the distributed connectivity pattern within regions defining the default network – the prototypical regions in our maps of distant connectivity – is not fully present in young children. An expanded analysis of the phenomenon demonstrated that childhood development is characterized by a general trend toward increases in functional connectivity across widely distributed regions conceptualized as the development of a ‘local to distributed’ organization [70]. These prior analyses suggest that detailed analysis of the development of local- and distant-connectivity profiles may provide important insights for both typical and atypical neurodevelopmental trajectories.

### Conclusions

Functional connectivity MRI was used to analyze network properties across the human brain introducing spatial distance information. We discovered that mapping regions based on whether they exhibit preferentially local versus preferentially distant functional connectivity at rest easily separates early sensorimotor, heteromodal association cortices and core regions of the default mode network. This observation reveals a parsimonious property of cortical network architecture that divides processing between many parallel systems characterized by extensive local processing and transmodal regions that serve as hubs connecting these local systems. As a practical application of our approach, metrics of connectivity profiles that reflect local and distributed connectivity can be made rapidly in individual participants. These metrics may thus have value for exploring individual differences both in relation to genetics and also in developmental neuropsychiatric disorders where atypical connectivity profiles may be present. More broadly, the observation that connectivity hubs fall within regions of estimated cortical expansion between monkey and humans [19], and also regions of late child development [71], reinforces the hypothesis that association areas make an important contribution to higher-order cognitive functions that are especially well developed in humans.

## Methods

### Participants

112 healthy young adults participated in MRI for payment. Table 1 shows the participant demographics. All participants had normal or corrected-to-normal vision and were right-handed, native English speakers. Participants were screened to exclude individuals with a history of neurologic or psychiatric conditions as well as those using psychoactive medications. Resting-state data from these participants have been reported previously [37], [65] and are openly available to the community upon request.

**Table 1:** Participant demographics.

	Data Set 1	Date Set 2	Composite Set	Task Data Set
**Sample size**	50 (21 Male)	50 (25 male)	100 (46 male)	12 (3 male)
**Mean age, yr (SD)**	22.1 (3.1)	22.3 (2.9)	22.2 (3.0)	22.1 (2.3)

Notes: SD = standard deviation. Data Sets 1 and 2 included data acquired during passive (rest) fixation. The Task Data Set included separate runs of fixation and continuous task performance (see text).

### Ethics Statement

Written informed consent was obtained in accordance with guidelines set forth by the institutional review board of Partners Healthcare Inc, and this research has been conducted according to the Declaration of Helsinki.

### MRI Acquisition Procedures

Scanning was performed on a 3 Tesla TimTrio system (Siemens, Erlangen, Germany) using the 12-channel phased-array head coil supplied by the vendor. High-resolution 3D T1-weighted magnetization prepared rapid acquisition gradient echo (MP-RAGE) images were acquired for anatomic reference (TR = 2530 ms, TE = 3.44 ms, FA = 7°, 1.0 mm isotropic voxels). Functional data were acquired using a gradient-echo echo-planar pulse sequence sensitive to BOLD contrast (TR = 2500 ms, TE = 30 ms, FA = 90°, 36–43 axial slices parallel to plane of the anterior commissure-posterior commissure, 3.0 mm isotropic voxels, 0.5 mm gap between slices). Head motion was restricted using a pillow and foam, and earplugs were used to attenuate scanner noise. During the functional runs, the participants fixated on a visual cross-hair (plus sign, black on white) centered on a screen for each of two runs (each run 7 min 24 sec; 148 time points). Participants were asked to stay awake and remain as still as possible. For the task condition, we used a data set previously reported in Buckner et al. [37]. Briefly, two runs of continuous task performance and two runs of fixation were acquired in twelve subjects (each run 5 min 12 sec; 104 time points). Participants decided whether centrally presented visual words represented abstract or concrete entities. Order of task was counterbalanced across participants. The visual stimuli were generated on an Apple PowerBook G4 computer (Apple, Inc., Cupertino, CA) using Matlab (The Mathworks, Inc., Natick, MA) and the Psychophysics Toolbox extensions [72]. Stimuli were projected onto a screen positioned at the head of the magnet bore.

### MRI Preprocessing

MRI analysis procedures were optimized for functional connectivity MRI (fcMRI) analysis [47] extending from the approach developed by Biswal et al. [45]. The first four volumes were removed to allow for T1-equilibration effects, followed by compensation of systematic, slice-dependent time shifts, motion correction and normalization to the atlas space of the MNI (SPM2, Wellcome Department of Cognitive Neurology, London, UK) to yield a volumetric time series resampled at 2 mm cubic voxels. Temporal filtering removed constant offsets and linear trends over each run while retaining frequencies below 0.08 *Hz*. Data were spatially smoothed using a 4 mm FWHM Gaussian blur (note that the effect of smoothing was explicitly considered in control analyses below).

Several sources of spurious or regionally nonspecific variance then were removed by regression of nuisance variables including: six parameter rigid body head motion (obtained from motion correction), the signal averaged over the whole-brain, the signal averaged over the lateral ventricles, and the signal averaged over a region centered in the deep cerebral white matter. Temporally-shifted versions of these waveforms also were removed by inclusion of the first temporal derivatives (computed by backward differences) in the linear model. This regression procedure removes variance unlikely to represent regionally specific correlations of neuronal origin. Of note, the global (whole-brain) signal correlates with respiration-induced fMRI signal fluctuations [47], [73], [74]. By removing global signal, variance contributed by physiological artifacts is minimized. Removal of signals correlated with ventricles and white matter further reduces non-neuronal contributions to BOLD correlations. Removal of global signal also causes a shift in the distribution of correlation coefficients such that there are approximately equal numbers of positive and negative correlations making interpretation of the sign of the correlation ambiguous [47], [75], [76]. This effect is not directly relevant to the current analyses as degree connectivity is computed based on the correlations that exceed a positive threshold. Finally, for computational efficiency, we down sampled the data to 4 mm isotropic voxels.

### Degree Connectivity Measures and Thresholds

The present study used fcMRI to map the local and distant degree of functional connectivity in the human brain. fcMRI measures intrinsic activity correlations between brain regions [45]–[47]. The method assumes that fcMRI is sufficiently constrained by anatomy to reveal informative estimates of connectivity properties. We have previously outlined the reasons for this assumption as well as the caveats and limitations of fcMRI [47]. For our present purposes it is important to make clear that fcMRI can reflect mono- and polysynaptic connectivity, correlations arising from common sources, and task-dependent dynamic functional coupling. Thus, it should not be considered a direct measure of anatomic connectivity. Nonetheless, fcMRI reflects, to a large degree, the statistical properties of anatomical connections and therefore provides a great deal of indirect information about human connectional anatomy.

Degree centrality (or degree) is a network measure that quantifies the number of links or edges connected to a node [27]. Here brain voxels (that sample small regions of cortex) are the nodes and positive correlations between voxels above certain strength are the links or edges in the graph. A computationally-efficient approach was used to map the degree of functional connectivity across the brain at the voxel level in a large number of individuals [37] taking into account topographical neighborhood information for the local and distant distinction.

Thus, we computed a variation of the classic degree centrality measure in graph theory (e.g., [27], [77]) by introducing physical distance restrictions in the whole-brain voxel-by-voxel functional connectivity network. The immediate neighborhood was taken into account to generate a local degree map and functional connectivity outside of this neighborhood was taken into account to generate a distant degree map. Different parameters of neighborhood threshold, in terms of radius sphere, were tested from which we choose 14 mm radius (approximately 3 voxels around target voxels) (see Figure S5). This distance threshold provides information about connectivity that is likely to reflect communication between local (nearby) areas and minimizes the correlations that reflect smoothing between adjacent voxels.

For these analyses, the time course of each voxel from the participant's brain defined within a whole-brain mask was correlated to every other voxel time course. As a result an *n*×*n* matrix of Pearson correlation coefficients was obtained, where *n* is the dimension of the whole-brain mask. The Pearson R, or product-moment correlation coefficient, computed in the *i*th row and *j*th column of this matrix is given by:
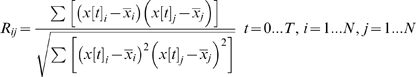
(1)where *t* is the frame count, 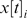 and 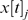 are the voxel intensities at the *i*th and *j*th voxel location respectively defined by the whole-brain mask at frame count *t* . The mean voxel intensity across all of the time points at the *i*th and *j*th voxel locations is given by 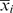 and 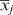 respectively. From the Pearson correlation coefficients, a map of degree connectivity was computed. We computed the local degree map by counting for each voxel the number of voxels above a correlation threshold of *r*>0.25 inside its neighborhood (defined as a 14 mm radius sphere), and for the distant degree map by counting for each voxel the number of voxels above the same threshold but outside the neighborhood. The r threshold was chosen to eliminate counting voxels that had low temporal correlation due to signal noise (see [37] for analysis of the effects of r threshold). No gap for counting voxels was included between the local and distant degree measures.

Finally, undirected and unweighted local and distant degree values were estimated for each voxel of the brain. The measure of degree connectivity for a voxel is given by:
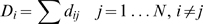
(2)where *j* is the inside or outside neighborhood voxels of the *i* depending on the measure of local or distant degree map. The degree connectivity map was then standardized by Z-score transformation so that maps across participants could be averaged and compared [37]. The conversion to Z-score does not influence the topography of the individual-participant maps but does cause the values in each participant's map to be comparable across subjects. In the present case, the Z-score transformation was computed separately for the local and distant degree connectivity maps. Moreover, these final Z-score degree maps were used to create the preferential and overlap maps. We refer here to preferential not as an absolute measure of number of links but rather as a relative measure of the overall topography differences (Local_Z-score_ minus Distant_Z-score_). Finally, the overlap map (Figure 4) was created by combining the local and distant Z-score maps after thresholding each of them at 1 SD in order to isolate only regions showing the strongest effects (see Figure S2 for other threshold criteria for the overlap map).

### Path lengths, physical cost and clustering coefficient

To situate our findings in the context of other well-known network measures, we computed in the same sample of subjects the following measures per node: path length (Figure 8; left column images), physical cost (Figure 8; center column images) and clustering coefficient (Figure 8; right column images), using matlabBGL (http://www.stanford.edu/~dgleich/programs/matlab_bgl/) and Boost Graph Library (http://www.boost.org/doc/libs/1_42_0/libs/graph/doc/index.html). For each subject, the functional connectivity MRI time series was first down-sampled from 4 to 8 mm voxel size for computational efficiency. We then formed a graph following the same principles as explained above but this time at 8 mm resolution. The path length of a node was calculated as the average path lengths from the node to all other nodes in the graph. Since each pair of connected nodes lie in physical space, we can assign a cost to each edge in the graph based on the physical Euclidean distance between the nodes in the brain. The physical cost of a node is therefore the average of the physical costs of all the edges of the node defined for each voxel as:
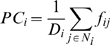
where the sum is over all the neighbors of voxel *i*, *D_j_* is the degree of voxel *i* and *f_ij_* is the Euclidean distance between voxel i and voxel j.

The clustering coefficient of a node is the proportion of all pairs of its neighbors that are directly connected to each other in the graph or, in other words, the number of links between the neighborhood vertices divided by the number of links that could possibly exist between them [31]. The measure of clustering coefficient for each voxel is given by:
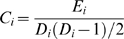
where *E_i_* is the actual number of links between the neighborhood vertices of voxel *i* and *D_i_* is the degree of voxel *i*. Observe that *D_i_(D_i_−1)/2* is the number of possible edges that could exist between the neighbors of voxel *i*.

### Control Analyses

**Figure 9:**
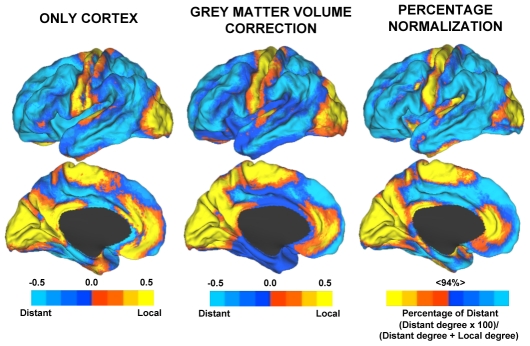
Preferential connectivity maps are similar in three control conditions. Map estimates of preferential local and distant connectivity do not notably change when subcortical structures are excluded from analysis, although several subtle differences are observed presumably arising from exclusion of distant thalamic, striatal, and cerebellar connections (left). Weighting the connectivity estimates based on the local gray matter volume also does not notably change the results (middle). Alternative normalization using percent normalization ([Distant Degree×100]/[Distant Degree + Local Degree]) shows qualitatively similar results as well (right).

Several control analyses were performed to explore the influences of processing decisions on the degree connectivity maps, to measure test-retest reliability, and to estimate the effects of local anatomy (Figures 9 and S1, S2, S3, S4, S5, S6, S7). We first evaluated the possible effects of distance threshold as well as spatial smoothing. Another concern is that the local degree connectivity measure along the midline is contaminated by strong correlations between homologous voxels across the left and right hemispheres. To explore this issue, we computed degree connectivity maps that masked the whole-brain (both hemispheres) as well as control analyses that restricted connectivity to within the hemisphere. We examined the influence of brain mask by comparing masks that involved the whole brain (including subcortical structures) versus a mask the included only cortical gray matter (excluding the basal ganglia, thalamus, and midbrain as well as the cerebellum). We also examined the influence of the local volume of grey matter in the neighborhood of the voxel (we refer to this as gray matter correction). For this analysis, the preferential map of local versus distant connectivity was estimated taking into account the number of grey matter voxels included in the search sphere rather than the absolute count, in order to appropriately weight regions that may contain less grey matter volume than others (such as voxels in the cortical/non-cortical interfaces). The normalized grey matter mask was obtained from SPM MarsBar toolbox (http://www.fil.ion.ucl.ac.uk/spm). Finally, we tested a second method to normalize degree maps in order to verify that our normalization procedure is not biasing our results using percentage normalization ([Distant Degree×100]/[Distant Degree+Local Degree]). While these control analyses do not represent an exhaustive set of possible processing variations, they bolster confidence that the major aspects of obtained results likely reflect properties of intrinsic functional connectivity that are not artifacts of anatomy or a specific processing decision.

### Visualization

Data were visualized on the cortical surface using the population-average, landmark- and surface-based (PALS) surface and plotted using Caret software [53], [78]. The PALS atlas is based on the PALS-B12 dataset from [79] and projects estimated areal boundaries from Broadmann's original architectonic scheme [80] to the surface. These area estimates are thus to be considered approximate. Reference boundaries for visuotopic-mapped areas (e.g., V1, V2v/d, V3) are based primarily on fMRI studies of human retinotopic mapping (e.g., [81]; see [53] for discussion).

## Supporting Information

Figure S1 Projections illustrate different visualization thresholds for preferential connectivity map. For comparison purpose, we display the preferential connectivity map at three distinct thresholds and include both left and right hemisphere projections to illustrate that its topography is qualitatively consistent across all variations.
(6.62 MB TIF)

Figure S2 Projections illustrate different visualization thresholds for regions that display both high local and high distant connectivity. Plotted in red are regions that show both high local and distant connectivity for three different thresholds: threshold 1 using normalized degree cutoff of Z-score>1.0, threshold 2 using normalized degree cutoff of Z-score>0.9 and threshold 3 using normalized degree cutoff of Z-score>0.8. As shown in the maps using threshold 1, the regions in both hemispheres that have high local and high distant connectivity at the same time are the regions that fall within the default network, such as the posterior cingulate, a region within the inferior parietal lobule, and the medial prefrontal cortex. Other regions, especially the superior parietal cortex, increase overlap while relaxing the threshold level. Left lateral and left medial view in threshold 1 are the same as Figure 4.
(3.86 MB TIF)

Figure S3 Volume display of local, distant and preferential map. The results were projected on cortical surface in the main paper to aid visualization of the cortical surface. For reference we plot here brain volume maps of local degree (A), distant degree (B) and the preferential connectivity map (C) that include subcortical and cerebellar regions.
(9.92 MB TIF)

Figure S4 Test-retest reliability for local and distant connectivity measures. The overall test-retest reliability of our approach was assessed with two different datasets of 50 participants each (dataset 1 and dataset 2). Degree maps for local (A) and distant (B) connectivity are highly correlated (r>0.90 in both cases). The figures on the left show the cortical projection in both samples and the graphs on the right the voxel-by-voxel correlation between Data Sets 1 and 2 for both analyses.
(4.29 MB TIF)

Figure S5 The effect of neighborhood distance threshold. In order to explore the influence of the neighborhood distance threshold for the analysis, we tested different sized spheres. The image shows maps for distance thresholds ranging from 6 to 18 mm in an example participant. A small radius such as 6 mm (approximately only one voxel around target voxel) yields a map that did not notably distinguish areal topography - the image is largely flat. This is likely because local correlations between very adjacent voxels dominate the computation. That is, there is little information about differential functional connectivity. Neighborhood sizes greater than 10 mm show clear topological differences along the cortex. However, neighborhood radii more than 10–14 mm are largely similar. As one extends the distant threshold further, the map eventually becomes equivalent to the distant-connectivity maps (data not shown). Therefore, we conservatively used a distance threshold of 14 mm for all analyses.
(2.70 MB TIF)

Figure S6 The effect of Gaussian smoothing. The influence of Gaussian smoothing was examined by comparing maps without spatial smoothing to the chosen 4 mm FWHM smoothing kernel. Comparison between degree measures with and without Gaussian smooth for local (A) and distant (B) degree maps reveals qualitatively similar results with both approaches. Some differences were noted with the predominant effect being the lower degree connectivity estimates obtained when no smoothing was applied (consistent with a reduction in signal-to-noise ratio).
(7.67 MB TIF)

Figure S7 Local connectivity along the midline. Estimates of local connectivity along the midline may be inflated because they include homologous right and left hemisphere regions. For this reason, it was important to evaluate the potential bias in overestimating local connectivity values along the midline. Here we show that maps that include local degree connectivity for only one hemisphere (right) are highly similar to the results in the main paper that involve both hemispheres (left). Thus contralateral correlations do not account for our observations in local connectivity.
(2.22 MB TIF)
