# Supplementary figures and images for: The Organization of Local and Distant Functional Connectivity in the Human Brain
Source: PLoS Comput Biol. 2010 Jun 10;6(6):e1000808. doi: 10.1371/journal.pcbi.1000808 (PMC2883589; doi:10.1371/journal.pcbi.1000808)

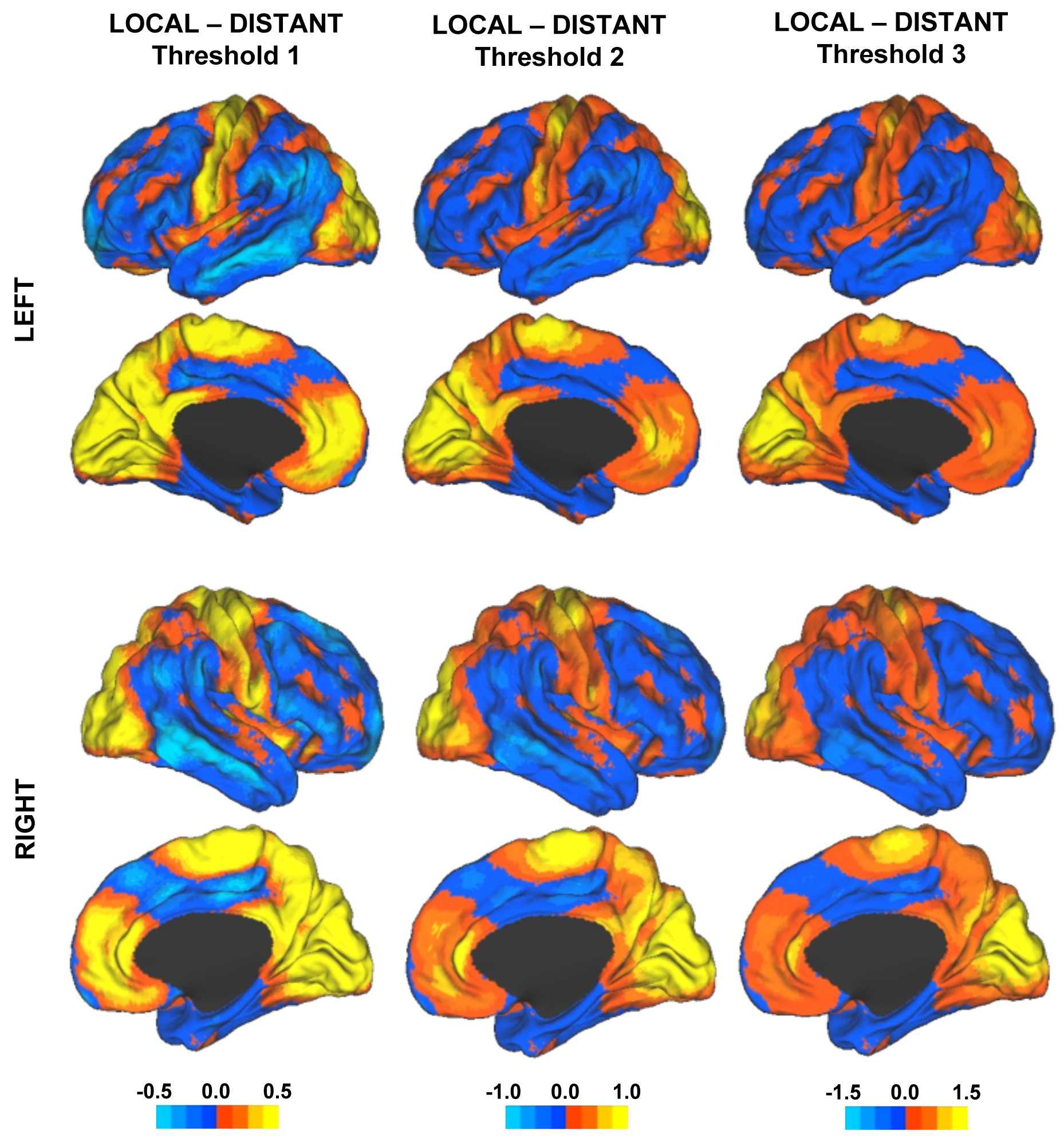

Supplement: Figure S1 — Projections illustrate different visualization thresholds for preferential connectivity map. For comparison purpose, we display the preferential connectivity map at three distinct thresholds and include both left and right hemisphere projections to illustrate that its topography is qualitatively consistent across all variations. (6.62 MB TIF) [file pcbi.1000808.s001.tif]

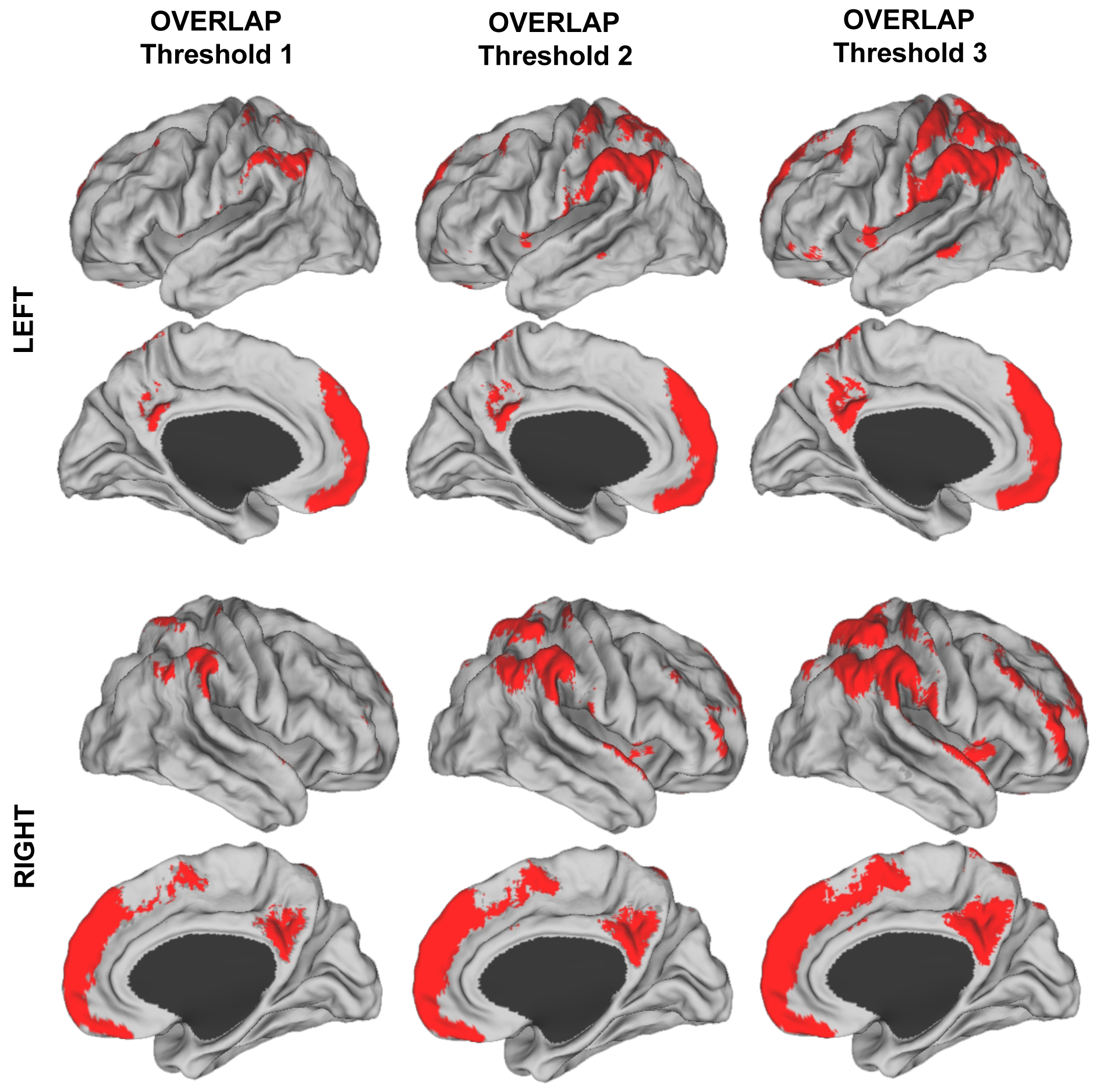

Supplement: Figure S2 — Projections illustrate different visualization thresholds for regions that display both high local and high distant connectivity. Plotted in red are regions that show both high local and distant connectivity for three different thresholds: threshold 1 using normalized degree cutoff of Z-score>1.0, threshold 2 using normalized degree cutoff of Z-score>0.9 and threshold 3 using normalized degree cutoff of Z-score>0.8. As shown in the maps using threshold 1, the regions in both hemispheres that have high local and high distant connectivity at the same time are the regions that fall within the default network, such as the posterior cingulate, a region within the inferior parietal lobule, and the medial prefrontal cortex. Other regions, especially the superior parietal cortex, increase overlap while relaxing the threshold level. Left lateral and left medial view in threshold 1 are the same as Figure 4. (3.86 MB TIF) [file pcbi.1000808.s002.tif]

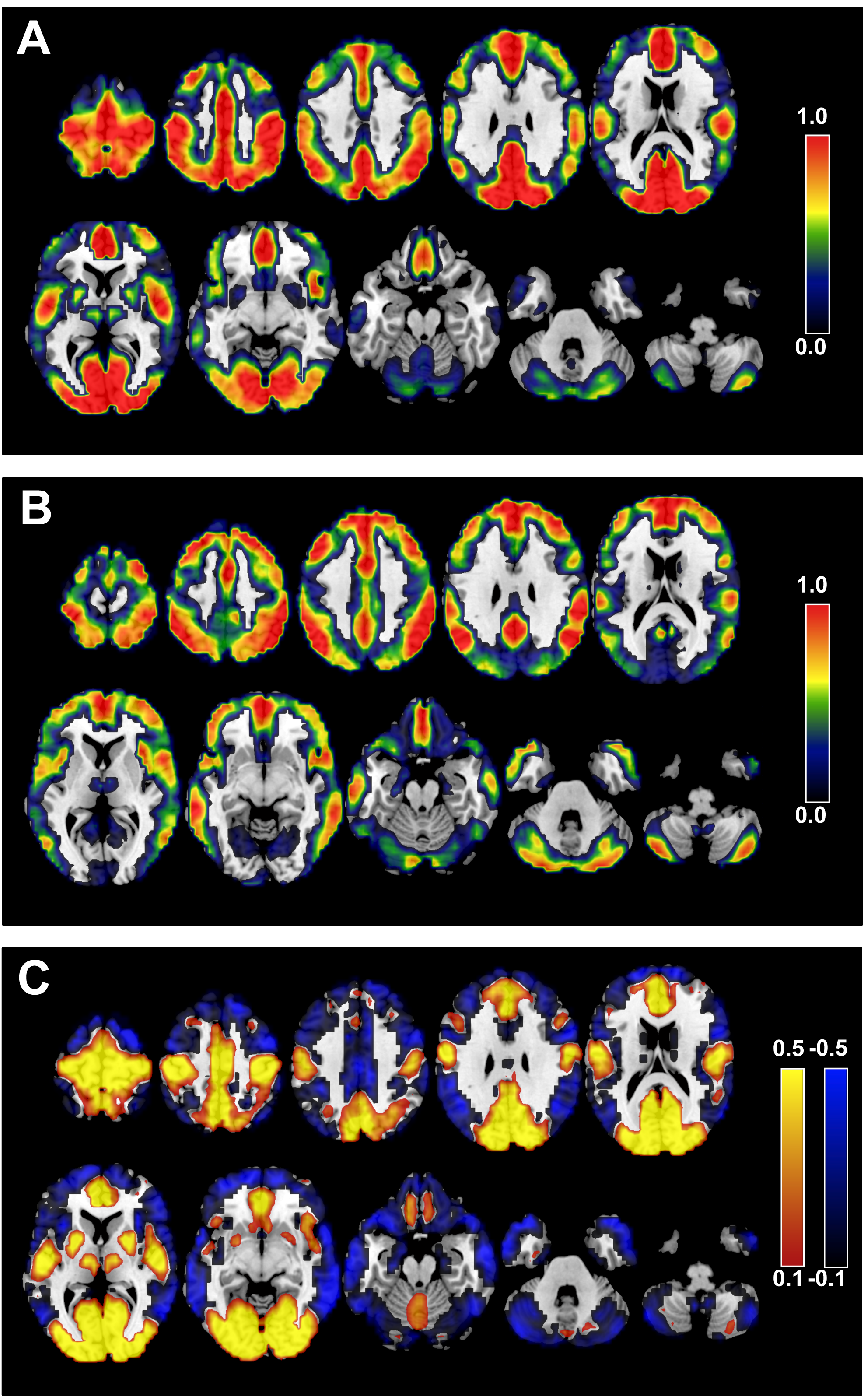

Supplement: Figure S3 — Volume display of local, distant and preferential map. The results were projected on cortical surface in the main paper to aid visualization of the cortical surface. For reference we plot here brain volume maps of local degree (A), distant degree (B) and the preferential connectivity map (C) that include subcortical and cerebellar regions. (9.92 MB TIF) [file pcbi.1000808.s003.tif]

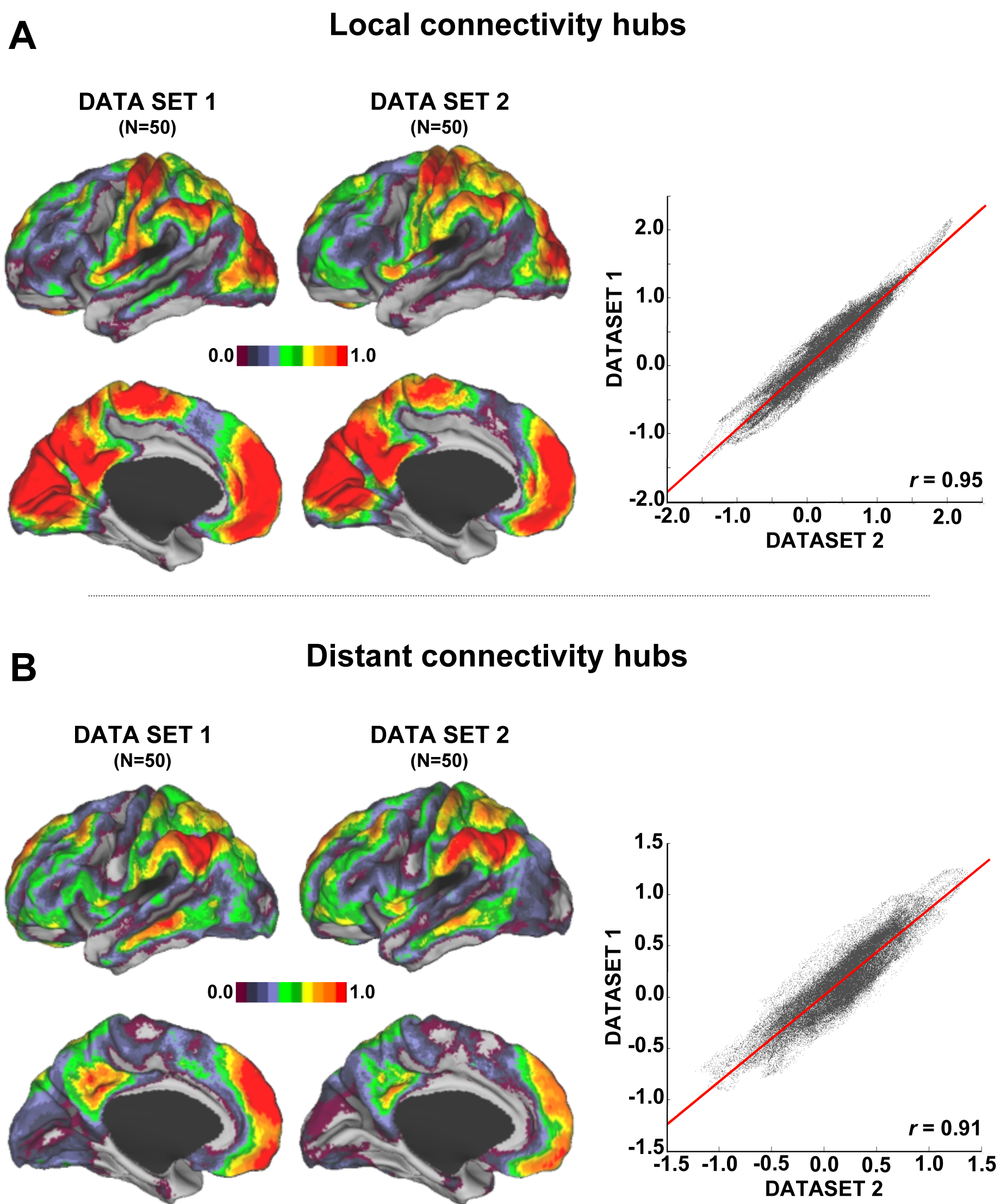

Supplement: Figure S4 — Test-retest reliability for local and distant connectivity measures. The overall test-retest reliability of our approach was assessed with two different datasets of 50 participants each (dataset 1 and dataset 2). Degree maps for local (A) and distant (B) connectivity are highly correlated (r>0.90 in both cases). The figures on the left show the cortical projection in both samples and the graphs on the right the voxel-by-voxel correlation between Data Sets 1 and 2 for both analyses. (4.29 MB TIF) [file pcbi.1000808.s004.tif]

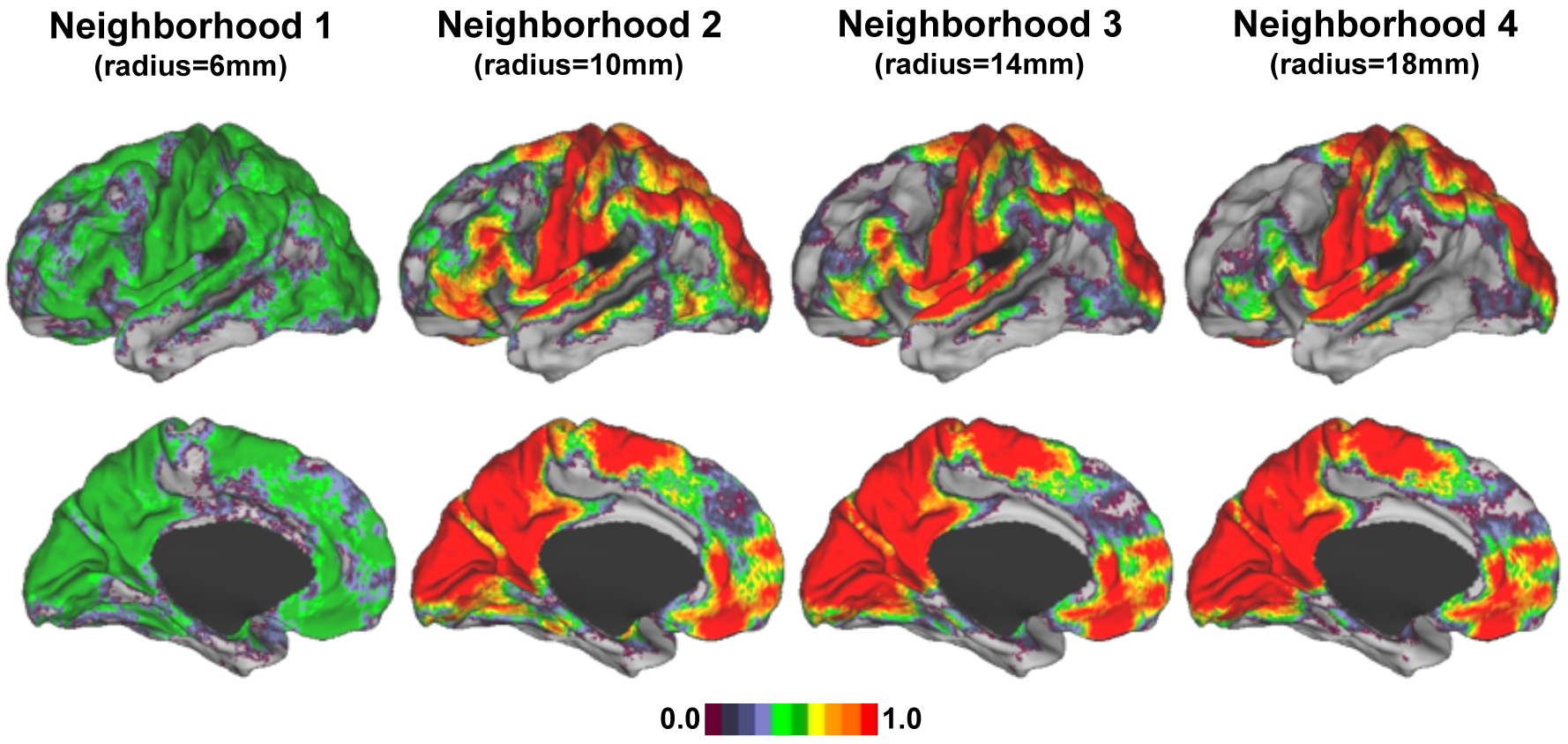

Supplement: Figure S5 — The effect of neighborhood distance threshold. In order to explore the influence of the neighborhood distance threshold for the analysis, we tested different sized spheres. The image shows maps for distance thresholds ranging from 6 to 18 mm in an example participant. A small radius such as 6 mm (approximately only one voxel around target voxel) yields a map that did not notably distinguish areal topography - the image is largely flat. This is likely because local correlations between very adjacent voxels dominate the computation. That is, there is little information about differential functional connectivity. Neighborhood sizes greater than 10 mm show clear topological differences along the cortex. However, neighborhood radii more than 10–14 mm are largely similar. As one extends the distant threshold further, the map eventually becomes equivalent to the distant-connectivity maps (data not shown). Therefore, we conservatively used a distance threshold of 14 mm for all analyses. (2.70 MB TIF) [file pcbi.1000808.s005.tif]

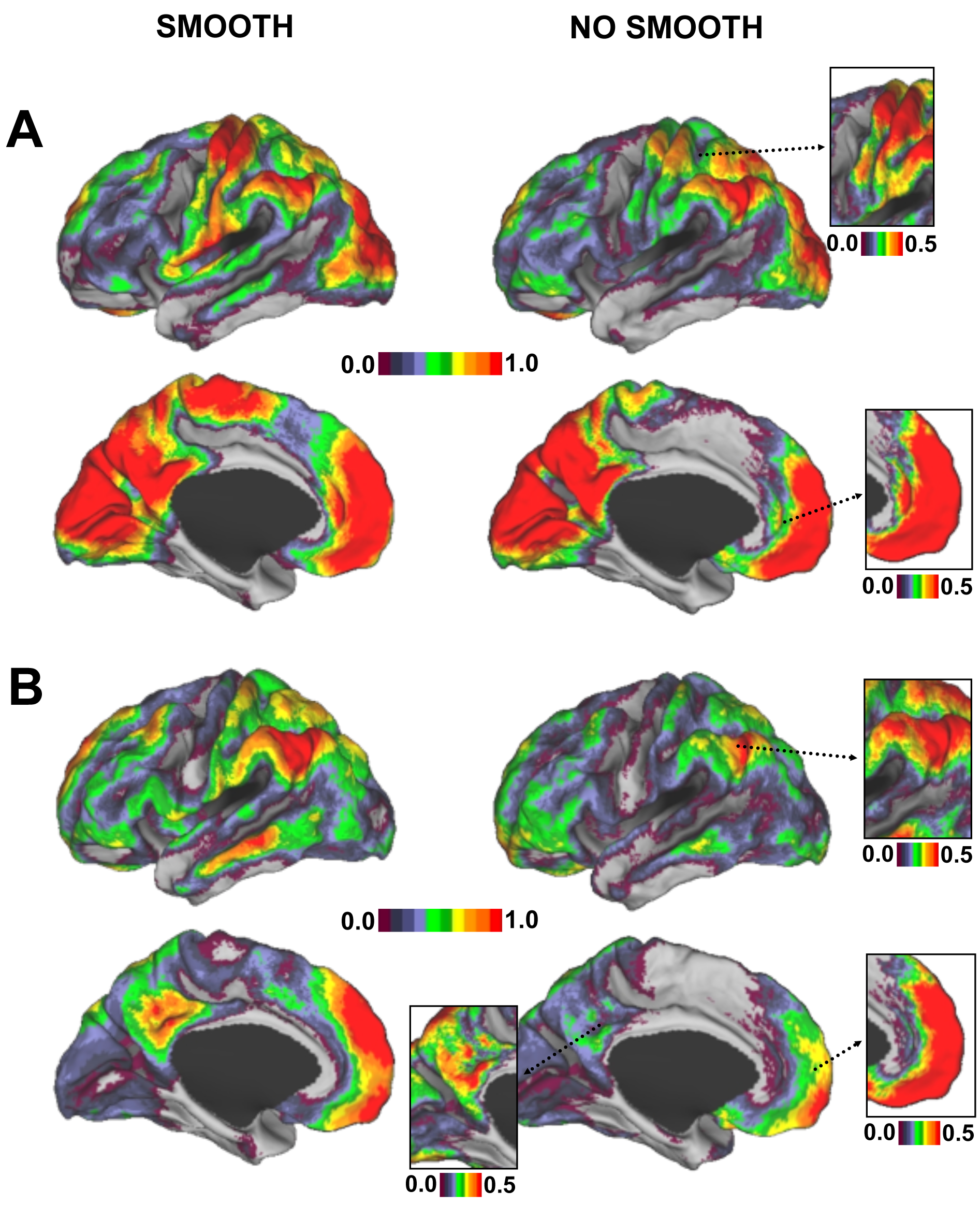

Supplement: Figure S6 — The effect of Gaussian smoothing. The influence of Gaussian smoothing was examined by comparing maps without spatial smoothing to the chosen 4 mm FWHM smoothing kernel. Comparison between degree measures with and without Gaussian smooth for local (A) and distant (B) degree maps reveals qualitatively similar results with both approaches. Some differences were noted with the predominant effect being the lower degree connectivity estimates obtained when no smoothing was applied (consistent with a reduction in signal-to-noise ratio). (7.67 MB TIF) [file pcbi.1000808.s006.tif]

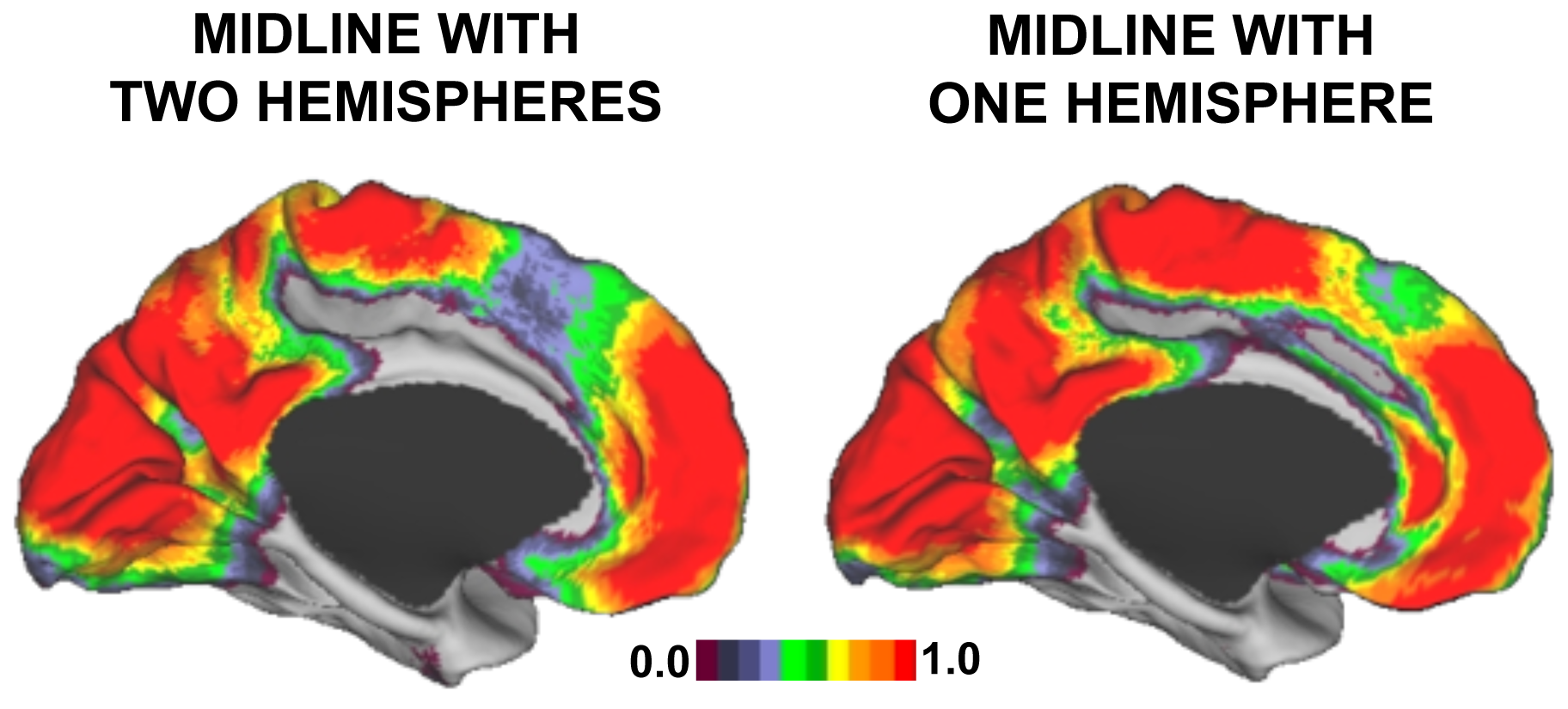

Supplement: Figure S7 — Local connectivity along the midline. Estimates of local connectivity along the midline may be inflated because they include homologous right and left hemisphere regions. For this reason, it was important to evaluate the potential bias in overestimating local connectivity values along the midline. Here we show that maps that include local degree connectivity for only one hemisphere (right) are highly similar to the results in the main paper that involve both hemispheres (left). Thus contralateral correlations do not account for our observations in local connectivity. (2.22 MB TIF) [file pcbi.1000808.s007.tif]
